# Addressing Social Injustice in Rural Japanese Healthcare: A Narrative Review Informed by Fraser’s and Sen’s Frameworks

**DOI:** 10.7759/cureus.100140

**Published:** 2025-12-26

**Authors:** Ryuichi Ohta

**Affiliations:** 1 Community Care, Unnan City Hospital, Unnan, JPN

**Keywords:** capability approach, foreign residents, health inequity, japan, older adults, recognition, redistribution, representation, rural health, social justice

## Abstract

Rural regions in Japan continue to experience significant health inequities despite universal health insurance coverage. These disparities disproportionately affect older adults, foreign residents, and socially isolated individuals, whose help-seeking is often delayed by structural, cultural, and informational barriers. This narrative review synthesizes empirical research, theoretical perspectives, and practice-based insights to clarify how social injustice manifests in rural Japanese healthcare and to identify strategies for more equitable care. Literature on rural health, health literacy, self-medication, mobility, and community participation was examined and interpreted through the frameworks of redistribution, recognition, and representation, as well as the capability approach. Across studies, persistent geographical barriers, transport limitations, and shortages of healthcare professionals restricted residents’ access to timely and appropriate care. Low health literacy, cultural differences, and weak social networks further hindered early help-seeking, particularly among foreign residents and older adults. Opportunities for participation in health decision-making were limited, reinforcing patterns of marginalization. Emerging community-based innovations-including non-profit-hospital collaborations, structured community dialogues, and digital consultation platforms-show potential to redistribute information, enhance cultural understanding, and strengthen residents’ capabilities to engage with the healthcare system. These findings suggest that achieving social justice in rural healthcare requires both structural reforms and capability-enhancing interventions that support meaningful participation by marginalized groups. Enhancing transportation, interpreter availability, community health worker programs, and co-designed communication pathways may contribute to a more inclusive and responsive rural healthcare system. This review provides a conceptual foundation for developing multilevel strategies to reduce inequities and promote social justice in rural Japan.

## Introduction and background

Rural healthcare systems worldwide face persistent structural challenges, including a limited healthcare workforce, reduced transportation options, and restricted access to specialized services. Japan, despite offering universal health insurance and a well-organized healthcare infrastructure, continues to experience significant inequities in its rural regions. These disparities disproportionately affect foreign residents, older adults, and socially isolated individuals, who frequently delay help-seeking until illness reaches an advanced stage. Prior studies have shown that foreign residents often confront language barriers, cultural unfamiliarity, and insufficient social networks, all of which hinder early access to care [1,2]. Older adults similarly face mobility limitations, digital illiteracy, and heightened vulnerability to misleading medical information, contributing to inappropriate self-medication and delayed clinical presentation [3-5]. These patterns demonstrate that universal health insurance does not automatically guarantee equitable access to timely and appropriate healthcare.

To understand why such inequities persist, analytical frameworks from social justice theory offer important insights. Nancy Fraser’s tripartite model-recognition, redistribution, and representation-highlights how cultural misrecognition, maldistribution of healthcare resources, and the political marginalization of disadvantaged groups collectively reproduce health disparities [6,7]. Amartya Sen’s capability approach complements this by emphasizing that justice requires more than the mere presence of services; it requires expanding individuals’ real freedoms and capabilities to seek, understand, and utilize healthcare [8]. When applied together, these frameworks reveal that inequities in rural Japan arise not only from structural barriers but also from deeper limitations on patients’ capabilities and participation in healthcare decision-making.

In rural Japanese communities, foreign residents often lack linguistic support mechanisms and community ties that enable effective help-seeking [1,2]. Older adults, who constitute a growing proportion of rural populations, commonly lose access to personal transportation due to age-related functional decline and must rely on limited public transportation options for hospital or pharmacy visits [3,9]. Meanwhile, national policies encouraging self-care and self-medication may unintentionally exacerbate risks among individuals with low health literacy [10,11]. These intersecting factors illustrate how multiple dimensions of social injustice shape rural health outcomes.

Recent innovations in rural Japan, including community dialogues, non-profit organization (NPO)-led initiatives, and digital platforms such as LINE-based anonymous medical consultations, demonstrate emerging pathways toward more equitable care [12-14]. These interventions redistribute information, elevate marginalized voices, and expand individuals’ capabilities for early help-seeking, aligning closely with both Fraser’s and Sen’s frameworks.

Although a growing body of narrative and scoping reviews has examined rural healthcare in Japan, most have conceptualized inequity primarily in terms of access, workforce distribution, or service utilization [1,4,5]. Such accounts, while informative, tend to treat social injustice descriptively and rarely engage with normative theories that explain how structural, cultural, and political dimensions of injustice interact in everyday healthcare experiences.

This review fills this gap by explicitly integrating Fraser’s framework of redistribution, recognition, and representation with Sen’s capability approach. This synthesis offers a novel analytical lens for understanding why health inequities persist in rural Japan despite universal health insurance-highlighting not only the maldistribution of resources but also the misrecognition of residents’ lived realities, limited participation in health-related decision-making, and constraints on individuals’ real capabilities to seek and use care. By applying this combined framework to empirical studies and community-based practices, the review advances understanding beyond access-focused or equity-descriptive reviews.

## Review

This review employed a narrative synthesis approach to integrate theoretical, empirical, and practice-based insights regarding social injustice in rural Japanese healthcare. Narrative review methodology was selected because the topic spans diverse domains-including public health, social justice theory, health policy, and community-based interventions-where heterogeneous evidence renders meta-analysis or systematic aggregation inappropriate.

Search strategy

A focused literature search was performed using PubMed, the primary international database for biomedical and public health research. The search was conducted in November 2025 using combinations of the following keywords and MeSH terms: “rural health,” “Japan,” “healthcare access,” “foreign residents,” “older adults,” “health inequity,” “help-seeking behavior,” “self-medication,” “social justice,” “Fraser,” and “capability approach.” Boolean operators (AND, OR) were applied to refine the search. No restrictions were placed on study design. Additional relevant articles were identified by screening the reference lists of key publications.

Eligibility criteria

Because this review aimed to integrate conceptual and empirical insights rather than conduct a statistical synthesis, the inclusion criteria were intentionally broad and grounded in conceptual relevance. Studies were considered eligible if they made meaningful contributions to understanding healthcare access, health literacy, help-seeking behavior, or the social determinants of health in rural Japanese contexts. Particular attention was given to research involving populations known to face disproportionate barriers in rural healthcare-such as foreign residents, older adults, and other marginalized groups whose experiences illuminate structural vulnerabilities.

In keeping with the review’s theoretical orientation, studies were also selected when they engaged with themes of recognition, redistribution, representation, or individual capabilities, as articulated in the frameworks of Fraser and Sen. A wide range of publication types-including peer-reviewed empirical studies, narrative and systematic reviews, policy analyses, and theoretical works-were included to ensure conceptual richness. Both English- and Japanese-language publications were eligible. Studies were excluded when they did not address social justice concerns, focused exclusively on urban settings, or lacked substantive relevance to healthcare access or community health dynamics in rural Japan.

Data extraction and synthesis

Data were extracted with attention to the core elements needed for a conceptual synthesis. For each study, information was gathered regarding research design, characteristics of the populations examined, documented barriers to healthcare access, help-seeking challenges, and the surrounding policy contexts. In addition, particular emphasis was placed on how each study contributed to understanding structural or experiential dimensions of social injustice. Fraser’s tripartite model of recognition, redistribution, and representation, along with Sen’s capability approach, served as guiding analytical categories throughout the extraction process.

During extraction, study design and inferential scope were explicitly noted to distinguish between descriptive associations, context-specific qualitative insights, and broader structural inferences. While all included studies informed the conceptual synthesis, their contributions were interpreted with attention to differences in methodological strength and explanatory reach.

The synthesis proceeded through an iterative narrative process. First, recurring patterns across studies were identified, revealing common challenges faced by foreign residents, older adults, and socially isolated individuals within rural communities. These patterns were then interpreted through the theoretical lenses of Fraser and Sen to clarify how social injustice manifests both structurally and in people’s lived experiences.

In this interpretive phase, evidence from studies with broader populations, larger samples, or convergent findings across multiple settings (e.g., national surveys, geographic analyses, and systematic or scoping reviews) was accorded greater inferential weight than single-site qualitative studies or individual case reports, which were used primarily to illustrate mechanisms and lived experiences. Where findings from studies of older adults or general rural populations were extended to discuss implications for foreign residents, such interpretations were treated as theoretically informed inferences rather than direct empirical conclusions, drawing on established literature on migration, language barriers, and social exclusion.

Finally, insights from practice-based evidence, such as rural NPO initiatives, community dialogues, and digital consultation tools, such as LINE-based services, were integrated to illuminate emerging solutions. These practice-based accounts were interpreted as illustrative and hypothesis-generating, complementing but not equating to empirical evidence at the population level. This approach enabled a holistic understanding of both the barriers and the innovative strategies shaping rural health equity in Japan.

Study selection and appraisal

Study selection followed an iterative, purposive process consistent with the narrative review methodology. Titles and abstracts identified through the PubMed search were screened for conceptual and contextual relevance to social injustice, healthcare access, help-seeking behavior, and rural health in Japan. Full texts were then reviewed to determine whether they contributed substantive empirical, theoretical, or practice-based insights aligned with the analytical frameworks of redistribution, recognition, representation, or capabilities. Studies were included based on relevance and explanatory value rather than methodological homogeneity.

Although the primary database searched was PubMed, the review also incorporated key theoretical works and interdisciplinary perspectives from social justice theory, sociology, and public policy through targeted citation tracking and screening of the reference lists of included articles. This approach was adopted to balance feasibility with conceptual breadth, recognizing that critical insights into rural health inequities may emerge outside strictly biomedical literature.

Formal risk-of-bias assessment or quality scoring was not undertaken, as this review aimed to synthesize heterogeneous evidence rather than compare intervention effects. However, study robustness was considered informally during synthesis by attending to study design, sample size, analytic transparency, and consistency of findings across different methodologies and settings. Greater interpretive weight was given to studies with clear methodological descriptions, theoretically informed analyses, or convergent findings across multiple sources.

Results

Study selection and characteristics

From the PubMed search, 24 articles were included in this narrative review. The studies spanned from 2004 to 2025 and were conducted mainly in rural prefectures such as Aomori, Hiroshima, Shimane, and Okinawa, with several nationwide ecological or claims-based analyses. Most designs were observational: approximately half were cross-sectional surveys or cross-sectional analyses within existing cohorts, with additional ecological and geographic information system (GIS) studies, one prospective cohort, one retrospective cohort, randomized controlled trial data, scoping reviews, and qualitative studies (Table 1).

**Table 1:** Included Studies on Rural Health Access and Health Literacy in Japan (2004–2025) This table provides a descriptive synthesis of the included studies examining geographic access, health service utilization, health literacy, and structural inequities among rural residents in Japan. Studies are arranged chronologically and include various rural contexts such as remote islands, mountainous regions, and depopulated municipalities, as well as selected national-level analyses. Key themes focus on access barriers, mobility, transport limitations, health literacy gaps, and social inequities across demographic groups and healthcare settings. HL: health literacy; LTC: long-term care; NPO: non-profit organization; NHI: National Health Insurance; IADL: instrumental activities of daily living; USC: usual source of care; GIS: geographic information system; RCT: randomized controlled trial; SES: socioeconomic status; HSB: help-seeking behavior; MUA: medically underserved area

Author (year)	Study design	Setting/region	Study population	Key themes (access, literacy, barriers, inequities)
Sakamoto et al. (2004) [15]	Cross-sectional survey	Aomori Prefecture, Japan	230 older adults (≥65); community-dwelling	Limited mental health access, transport barriers, low literacy, stigma, low professional help-seeking
Matsumoto et al. (2013) [16]	Geographic accessibility analysis	Hiroshima Prefecture, Japan	7,374 dialysis patients; full geographic data	Significant geographic disparities in travel time; rurality strongly linked to poor access; administrative “rural” labels fail to capture true low-access areas
Lagergren and Kurube (2014) [17]	Cross-sectional ecological comparison	Four Japanese municipalities: 2 urban and 2 rural	Older adults ≥ 65; municipal LTC certification and service-use data	Rural areas have fewer LTC facilities and home-help services despite higher care needs; long distances, weak public transport, workforce shortages, and reliance on informal care create spatial inequality
Hamano et al. (2016) [18]	Cross-sectional study	Shimane Prefecture, Japan	876 adults aged 40–74 receiving health check-ups	Non-drivers face significant access limitations due to weak public transport, increasing mental health risk; mobility deprivation creates structural inequity
Hamano et al. (2017) [19]	Cross-sectional study	Shimane Prefecture, Japan	710 adults aged 40–74; NHI health check-up participants	Longer distance to dental clinics and lack of transport for non-drivers; physical access explains disparities; distance is associated with tooth loss risk
Uemura et al. (2018) [20]	Randomized controlled trial	Imizu City, Toyama, Japan	84 community-dwelling older adults (≥65 years)	Limited access to preventive health education; rural older adults face information gaps; active-learning program improves health literacy
Yamaguchi et al. (2018) [21]	Cross-sectional community survey	Nichinan Town, Tottori, Japan	474 adults ≥ 40 years; household meal preparers	Long travel distance to food shops, limited transport, IADL decline, high isolation; food access barriers mirror rural medical access inequities
Namiki and Kobayashi (2018) [22]	Retrospective cohort study	Yonaguni Island, Okinawa, Japan	6,197 clinic visits from all residents	Extreme geographic isolation; dependence on a single clinic; high cost/time for off-island care; structural limitations in medical resources and emergency services
Manabe et al. (2019) [23]	Cross-sectional survey	Rural clinics in Gifu (7), Niigata (3), Shizuoka (2), Kochi (5), Japan	321 residents (≥20 years); younger (20–60s) vs. older (≥70)	Physician shortages; rotating doctors; limited hours; generational differences in expectations; digital divide; transport challenges in rural/mountain areas
Kaneko et al. (2019) [24]	Prospective open cohort study	14 isolated islands, Okinawa Prefecture, Japan	12,238 residents; clinic users from islands with ≥26% older adults	Single clinic per island; no inpatient beds; reliance on boat/air travel; high travel cost/time; limited specialist access; SES disadvantage; geographic inequity
Ohta et al. (2021) [25]	Cross-sectional study	Rural clinics in Unnan City, Shimane Prefecture, Japan	169 older adults (≥65 years) attending two rural clinics	Long distance to hospital; limited medical resources; sparse public transport; geographical isolation; high prevalence of low health literacy; SES disadvantage; social isolation; structural barriers affecting help-seeking behavior
Kaneko et al. (2021) [26]	Systematic scoping review	International literature	Studies using rurality indices	Geographic access central to rurality; transport limitations; lack of public transport; physician shortages; inconsistent definitions of rural areas
Ohta et al. (2021) [27]	Cross-sectional survey	Unnan City, Shimane Prefecture, Japan	Rural residents ≥ 65 years; cognitively intact; n = 834	High reliance on primary care due to limited medical facilities; low availability of family/community support resources. Low health literacy, limited information-seeking behaviors
Ohta and Sano (2022) [28]	Cross-sectional study	Unnan City, Shimane Prefecture, Japan	Older adults aged ≥65; n = 834 respondents (78.2% response rate)	Mobility declines and a lack of transport restrict access. Variable and generally low health literacy; SES and frailty-related mobility limits, reduced family support
Ohta et al. (2023) [29]	Thematic analysis	Unnan City, Shimane Prefecture, Japan	81 residents including community leaders, NPO staff, local officials, and healthcare workers	Decline in help-seeking due to isolation and cultural/organizational barriers rather than distance. Difficulty understanding/using health information. Weak community ties, administrative overload on local groups, and distrust in government communication
Ohta and Sano (2023) [30]	Case report	Unnan City, Shimane Prefecture, Japan	72-year-old woman, living alone, non-driver, low income	Severe geographical/transportation barriers for non-drivers; difficulty attending regular follow-up. Low health literacy; difficulty accessing digital information. Social isolation, financial constraints, and self-aging lead to delayed care
Ohta and Sano (2023) [13]	Grounded theory/ethnography	Unnan City, Shimane Prefecture, Japan	112 rural residents from two communities	Few hospitals/clinics, a mental distance from physicians, and limited public transport. Residents learn about HSB through peer experiences; they lack formal channels for health information
Ohta et al. (2024) [31]	Thematic analysis	Unnan City, Shimane Prefecture, Japan	57 community workers (mean age 65.4 years) active in local self-governments	Loss of mobility, scarce public transport, and lack of escorts for clinic visits. Privacy concerns reduce information sharing; difficulty accessing health information; medical staff are perceived as distant-isolation, declining mutual support, youth outmigration, and hesitation to seek help
Morishita-Suzuki and Watanabe (2024) [32]	Cross-sectional study	Tsumagoi Village, Gunma Prefecture, Japan	Community-dwelling older adults aged ≥75 (n = 411)	The “multisources” group had the highest HL. 16.8% reported no information sources; internet use was extremely low (2.7%); weak family/neighbor information networks
Nakamura et al. (2024) [33]	GIS-based simulation study	Entire Japan	Geographic units across all medical service areas	Mountainous and inland rural regions will experience the greatest increase in new MUA. Reduced facility access is expected to worsen chronic and acute care outcomes
Kaneko et al., 2024 [34]	Cross-sectional survey	Japan	General adults aged 20–74 years	Access to primary care remained stable across rurality levels; USC associated with better patient experience
Ohta et al., 2024 [14]	Grounded theory	Unnan City, Shimane Prefecture, Japan	Rural residents using the online consultation platform	Geographic isolation and physician shortage led to delayed care-seeking; mistrust of physicians reduced utilization; low health literacy enabled misinformation; anonymous dialogue improved psychological safety and timely help-seeking
Kaneko et al., 2025 [35]	Scoping review	National-level review across Japan	Studies covering regional health systems	Structural geographic barriers; specialist maldistribution; delayed emergency care; limited specialist access; broader scope of practice for rural physicians
Shibata and Kaneko, 2025 [36]	Ecological study	Japan, all 47 prefectures	Home-based medical care users; service utilization	Higher rurality strongly associated with lower use of home-based medical care: lower rates of regular visits, urgent visits, end-of-life care, and death certification

Across the dataset, 13 of 24 studies focused primarily on older adults (typically ≥65 years), while others examined general rural residents, dialysis patients, home-care users, or community workers in depopulated or geographically isolated areas. Almost all studies explicitly addressed rural-urban differences or intra-rural disparities in access to health services, help-seeking behavior, or health outcomes. Using Fraser’s framework and Sen’s capability approach, each study was mapped to dimensions of redistribution (e.g., spatial distribution of services, transport, and workforce), recognition (e.g., health literacy, stigma, and cultural norms), and representation (e.g., participation of residents and community actors in shaping care).

Inclusion, health, and social justice among diverse rural residents

The included studies revealed that structural features of rural Japan-population aging, depopulation, mountainous terrain, and island geography-systematically constrained residents’ access to healthcare and social care resources. Geographic analyses showed that travel time and distance to facilities were strongly patterned by rurality: dialysis patients, dental patients, and residents requiring long-term care in remote communities faced substantially longer travel times and fewer facility choices than those in urban or semi-urban areas [16,18,19,21,22,34,36]. These constraints limited individuals’ capability to choose among providers and to receive timely care, even when they were nominally covered by national health insurance.

Ecological comparisons of urban and rural municipalities suggested a mismatch between needs (e.g., higher prevalence of disability, frailty, or need for long-term care) and provision (e.g., fewer formal services and lower availability of home-care or dental services) in rural settings [15,33,36,37]. In several studies, rural residents were more likely to live in areas with high care needs but relatively low service density, indicating a form of maldistribution in Fraser’s sense of redistribution [17,25,26,28,32,34]. At the same time, qualitative and mixed-method studies highlighted that residents’ help-seeking behaviors were shaped by norms of self-reliance, limited awareness of services, and expectations that minor or moderate symptoms should be managed within the family or neighborhood rather than through formal care [13,31].

Although not all included studies focused explicitly on foreign residents, the mechanisms they described-geographic isolation, limited facility choice, scarcity of primary care and community health professionals, and dependence on informal social networks-are particularly likely to disadvantage people who lack strong local ties, including migrants and socially isolated residents [2,38]. In Sen’s terms, many rural inhabitants had resources on paper (insurance coverage, nominal facility access) but lacked the real freedoms and conversion factors (transport, information, and social support) required to translate those resources into effective healthcare utilization.

Older patients’ challenges in rural health care: access, literacy, and help-seeking

A large proportion of the included studies concentrated on older adults, revealing intersecting injustices in redistribution, recognition, and representation.

Physical access and transport: Multiple studies showed that loss of driving ability among older adults in rural areas led to “transport-poor” patients who were heavily dependent on family members, neighbors, or infrequent public transport to access hospitals, clinics, and pharmacies [1,18,19,23,26]. Non-drivers living in low-altitude rural areas with limited public transport had significantly higher odds of depressive symptoms, suggesting that restricted mobility and social participation were closely linked to mental health [15,32,33]. Distance to dental clinics was independently associated with tooth loss among older residents, indicating that geographic barriers extended beyond hospitals to preventive and oral health services as well [19,23,34].

Health literacy, self-medication, and vulnerability to misinformation: Several studies examined self-medication, food environments, and information environments among older adults. Cross-sectional surveys found that older rural residents frequently relied on over-the-counter (OTC) medications and supplements for mild symptoms, often without adequate understanding of potential side effects or drug interactions [3,17,28]. Low health literacy and limited access to high-quality health information increased the risk of inappropriate self-medication, particularly in the context of national policies promoting self-care and OTC use.

Studies of food access and nutrition showed that “food deserts” existed in some rural communities, where older adults with declining instrumental activities of daily living (IADL) had difficulty obtaining nutritious food [16,20,21]. These environmental constraints intersected with health literacy gaps, amplifying vulnerabilities to chronic disease and medication mismanagement. From a recognition perspective, policies that assume older adults can safely self-manage their health may misrecognize their actual capabilities and overlook age-related declines in cognition, mobility, and digital literacy.

Help-seeking behaviors and psychosocial factors: Research on help-seeking behaviors among older rural residents indicated that many individuals preferred to endure symptoms or confide only in close family rather than consult healthcare professionals [3,15,21,22,27,39]. In one study, the proportion of older adults with depressive symptoms who sought professional help was very low, and stronger depressive symptoms were paradoxically associated with decreased help-seeking [15]. Social isolation, shrinking friendship networks, and stigma around mental illness were repeatedly described as barriers.

At the same time, some studies suggested that regular participation in health check-ups, structured exercise programs, or community activities could improve physical function, cognitive status, and health literacy, thereby indirectly supporting earlier help-seeking [20,25]. These findings illustrate how capabilities are dynamic: when older adults gain mobility support, information, and social connection, their freedom to seek timely care and participate in health decision-making increases.

Driving rural health promotion through non-profit action, dialogue, and collaboration

A third group of studies and qualitative reports focused on community-based and organizational solutions to rural health inequities, including the work of NPOs, community organizations, and family physicians engaged in community-oriented primary care [13,14,31].

Community participation and dialogue: Grounded theory and thematic analyses of rural community initiatives showed that structured dialogues among residents, family physicians, and local officials could enhance mutual understanding of health problems, including loneliness, multimorbidity, and difficulty accessing care [13,14,29]. In these studies, community meetings and “community health dialogue” served as spaces where older adults and other residents articulated their concerns, shared experiences, and co-developed ideas to improve help-seeking and service use. These processes directly addressed Fraser’s dimension of representation, enabling marginalized residents to participate in shaping local health agendas rather than remaining passive recipients of services.

NPO-hospital collaboration and digital platforms: Several recent qualitative and mixed-method studies described collaborations between rural hospitals and NPOs that leveraged both face-to-face and digital approaches. Examples included NPO-facilitated community forums where residents, municipal officers, and healthcare workers discussed barriers to care and co-created solutions [31]; LINE-based or other online consultation platforms allowing residents to ask anonymous health questions to medical professionals, revealing “hidden” concerns that might not surface in clinic visits or public meetings [14]; and community workers and volunteers acting as intermediaries who support older adults in interpreting health information, navigating services, and participating in community activities [13].

These interventions were interpreted as forms of redistribution (sharing professional expertise and information more equitably across the community), recognition (valuing the lived experiences and narratives of older and marginalized residents), and representation (incorporating community input into program design and local policy discussions). From a capability perspective, they expanded residents’ real opportunities to seek help early, clarify doubts, and engage with the healthcare system on more equal terms.

System-level perspectives on rural-urban disparities: Scoping and ecological studies at the national level synthesized evidence on rural-urban disparities in physician distribution, hospital bed availability, home-based medical care, and usual source of care [24,26,34,36]. These analyses underscored that individual-level interventions must be situated within broader structural reforms: rural areas with high proportions of older adults often had fewer physicians and weaker primary care infrastructure, leading to higher reliance on hospitals and emergency care. Narrative syntheses argued that sustainable solutions require multilevel action: strengthening primary care and home-based care, investing in transportation and digital infrastructure, and institutionalizing mechanisms for community participation in health planning.

Discussion

This narrative review demonstrates that social injustice in rural Japanese healthcare emerges from the intersection of structural constraints, cultural norms, and limited opportunities for meaningful participation among marginalized residents. Across the reviewed studies, recurring disparities reflected Fraser’s dimensions of redistribution, recognition, and representation. At the same time, Sen’s capability approach illuminated how these structural and cultural barriers restrict individuals’ fundamental freedoms to seek and receive timely healthcare.

Redistribution inequities were consistently documented. Rural communities-particularly remote islands, mountainous regions, and depopulated towns-exhibited persistent shortages of healthcare providers, limited transportation infrastructure, and reduced access to essential services such as dental care, home-based care, and long-term care [17,19,36]. Older adults who had lost driving ability and foreign residents without cars or local support networks were especially affected [40]. Even with universal insurance coverage, access to services was unevenly distributed, and the effective availability of care was constrained by geography, transportation, and workforce maldistribution [41]. These findings align with earlier evidence indicating that universal financial coverage alone cannot eliminate spatial or logistical inequities in healthcare systems [42,43].

At the same time, recognition injustices contributed to delayed help-seeking and inappropriate self-management. Many rural residents-particularly older adults and foreign residents-experienced low health literacy, limited access to trustworthy information, and cultural norms favoring stoicism or family-based care [44]. National policies promoting self-medication risked exacerbating disparities when implemented without accounting for differences in digital literacy, cognitive decline, or cultural background [45]. Studies also showed that foreign residents face additional challenges stemming from language barriers, cultural unfamiliarity, and lack of social networks, often leading to advanced presentations of serious illnesses such as cancer or heart failure [46]. These patterns reflect misrecognition, in which policies assume capabilities that marginalized groups may not possess.

Representation-the political and participatory dimension of justice-emerged as a critical but under-addressed domain. Marginalized groups, including foreign residents, socially isolated older adults, and low-income households, rarely participate in community decision-making about health services [47]. Consequently, policies may overlook their needs or unintentionally reinforce inequities [48,49]. The review found that participatory approaches-such as community dialogues, NPO-led forums, and digital platforms enabling anonymity-can uncover hidden concerns, empower residents, and inform local health planning. These initiatives suggest promising avenues for enhancing participatory justice in rural settings.

Sen’s capability approach adds an essential layer to this analysis by emphasizing that justice requires expanding individuals’ actual opportunities to access care [8]. Even when services are available, many rural residents lack the conversion factors-transportation, information, social support, and trust-to translate these resources into effective healthcare use [50]. Notably, several studies have shown that when mobility support, information access, and community engagement are improved, help-seeking behavior and health literacy increase [51,52]. These findings underscore the dynamic and interdependent nature of capabilities and structural supports.

Together, the evidence suggests that addressing social injustice in rural Japan requires multilevel strategies. Structural reforms-such as improving transportation, redistributing healthcare workers, and enhancing interpreter services-must be coupled with capability-enhancing interventions, including health literacy initiatives, digital platforms for anonymous consultation, and community-led dialogue for co-designing services. The role of NPOs and community organizations appears especially valuable, as they can bridge gaps between residents and formal medical institutions, providing culturally sensitive support while amplifying marginalized voices.

Among these strategies, structural interventions such as transportation support, redistribution of primary care resources, and interpreter services are supported by relatively consistent evidence across geographic analyses and population-based studies, suggesting higher feasibility and scalability within existing policy frameworks [42-44]. In contrast, NPO-led collaborations, community dialogues, and digital consultation platforms should be regarded as promising but under-evaluated innovations, currently supported mainly by qualitative and single-site studies [31].

Several limitations must be acknowledged. First, the narrative review approach, while suited for synthesizing heterogeneous evidence, does not provide the systematic rigor or replicability of formal systematic reviews. Publication bias may have influenced the scope of available studies, particularly regarding underreported experiences of foreign residents or undocumented migrants in rural settings. Second, the review relied primarily on studies indexed in PubMed and may not have captured relevant literature from sociology, anthropology, public policy, or regional studies that examine rural Japanese communities from non-medical perspectives. This may have limited the theoretical richness and cross-disciplinary integration of findings. Third, most included studies were cross-sectional, qualitative, or ecological in nature, limiting causal inference. Few studies directly examined interventions aimed at reducing health inequities, and fewer still evaluated long-term outcomes of community-based or NPO-led initiatives. As a result, evidence regarding effective strategies remains largely descriptive and context-specific. Fourth, the experiences of foreign residents-one of the key populations in this review-were only tangentially represented in the available literature. Many studies focused on older adults or general rural populations, leaving gaps in understanding the unique health-seeking patterns and barriers faced by migrants. The review, therefore, extrapolates from related evidence, which may not fully capture the heterogeneity of foreign residents’ circumstances. Finally, because some insights in this review derive from experiential knowledge and practice-based accounts, they may reflect contextual nuances that differ across rural regions. Rural Japan is not monolithic; geographic, cultural, and administrative variations limit the generalizability of specific observations or proposed solutions.

## Conclusions

This narrative review shows that social injustice in rural Japanese healthcare arises from intersecting deficits in redistribution, recognition, and representation, which together limit residents’ capabilities to access and use healthcare. Structural barriers-such as geographic isolation, transport limitations, and workforce shortages-combine with cultural and informational inequities to disproportionately burden older adults, foreign residents, and socially isolated individuals. Fraser’s and Sen’s frameworks clarify how these inequalities are produced and maintained across multiple levels of the healthcare system. Emerging innovations, including community dialogues, NPO-hospital collaborations, and anonymous digital consultation platforms, demonstrate promising approaches to address these gaps. Such interventions can redistribute information, improve cultural recognition, and expand opportunities for resident participation. Moving toward a more just rural healthcare system will require investment in both structural resources and participatory mechanisms that empower marginalized groups. Policies grounded in co-design, culturally responsive communication, and cross-sector collaboration are especially crucial. By integrating structural reforms with capability-enhancing strategies, Japan can build a rural healthcare system that is more equitable, inclusive, and responsive to diverse community needs. In addition, the inclusion of practice-based and experiential insights introduces the possibility of author positionality and interpretive bias; while these perspectives enrich contextual understanding, the synthesis reflects a reflexive, theory-informed interpretation rather than a neutral aggregation of evidence.
